# Acute Respiratory Failure in Children: A Clinical Update on Diagnosis

**DOI:** 10.3390/children11101232

**Published:** 2024-10-12

**Authors:** Beatrice Panetti, Ilaria Bucci, Armando Di Ludovico, Giulia Michela Pellegrino, Paola Di Filippo, Sabrina Di Pillo, Francesco Chiarelli, Marina Attanasi, Giuseppe Francesco Sferrazza Papa

**Affiliations:** 1 Pediatric Allergy and Pulmonology Unit, Department of Pediatrics, University of Chieti, 66100 Chieti, Italy; beatrice.panetti@studenti.unich.it (B.P.); ilaria.bucci@studenti.unich.it (I.B.); armando.diludovico@studenti.unich.it (A.D.L.); difilippopaola@libero.it (P.D.F.); sabrinadipillo@libero.it (S.D.P.); chiarelli@unich.it (F.C.); 2 Department of Neurorehabilitation Sciences, Casa di Cura Igea, 20144 Milan, Italy; g.pellegrino@casadicuraigea.it (G.M.P.); g.sferrazza@casadicuraigea.it (G.F.S.P.)

**Keywords:** acute respiratory failure, children, lung function, lung ultrasound, chest X-ray, chest computed tomography, lung magnetic resonance imaging, biomarkers, artificial intelligence

## Abstract

Acute respiratory failure (ARF) is a sudden failure of the respiratory system to ensure adequate gas exchanges. Numerous clinical conditions may cause ARF, including pneumonia, obstructive lung diseases (e.g., asthma), restrictive diseases such as neuromuscular diseases (e.g., spinal muscular atrophy and muscular dystrophy), and albeit rarely, interstitial lung diseases. Children, especially infants, may be more vulnerable to ARF than adults due to anatomical and physiological features of the respiratory system. Assessing respiratory impairment in the pediatric population is particularly challenging as children frequently present difficulties in reporting symptoms and due to compliance and cooperation in diagnostic tests. The evaluation of clinical and anamnestic aspects represents the cornerstone of ARF diagnosis: first level exams (e.g., arterial blood gas analysis) confirm and evaluate the severity of the ARF and second level exams help to uncover the underlying cause. Prompt management is critical, with supplemental oxygen, mechanical ventilation, and the treatment of the underlying problem. The aim of this review is to provide a comprehensive summary of the current state of the art in diagnosing pediatric ARF, with a focus on pathophysiology, novel imaging applications, and new perspectives, such as biomarkers and artificial intelligence.

## 1. Introduction

Acute respiratory failure (ARF) is a critical condition that poses significant clinical challenges. ARF is a prominent cause for admission in pediatric intensive care units (PICU) [1], and represents a significant contributor to both morbidity and mortality [2]. ARF is one of the most prevalent causes of cardiopulmonary arrest in the pediatric population.

ARF diagnosis and management requires a sound understanding of its pathophysiology and clinical presentation. Critical clinical signs include: rapid and labored breathing, abnormal breathing patterns, changes in alertness, and abnormal oxygen and carbon dioxide levels in the blood. Pulse oximetry is a key tool for monitoring oxygen saturation, but it is important to remember that certain circumstances can lead to inaccuracies.

For example, high carboxyhemoglobin levels can affect SpO_2_ measurements, intravenous colorants like methylene blue may artificially lower SpO_2_ [3], and conditions such as shock, hypovolemia, or hypothermia can reduce blood flow, weakening the signal and causing inaccurate readings [4]. Additionally, ARF can also progress to pediatric acute respiratory distress syndrome (PARDS) in children, as in severe cases of Coronavirus Disease 2019 (COVID-19) [5], characterized by heterogeneous damage to the alveolar–capillary barrier, inducing an overly robust inflammatory reaction as well as injury to the pulmonary endothelium [6]. The mortality rate for PARDS is lower than that for adults [7], although it remains a significant concern for pediatric healthcare workers [8].

Despite chest X-ray (CXR) being the traditional method for evaluating children with acute respiratory distress and failure [9], lung ultrasound (LUS) is recognized as a valuable alternative in pediatric care, and the relevance of its use is particularly important in light of concerns surrounding radiation exposure in children [10,11,12]. While LUS has been more extensively studied and applied in adults (such as in the BLUE protocol [13]), its use in the pediatric setting is gaining increased attention [14]. The key advantages of LUS include safety, portability, and suitability for real-time bedside monitoring [15]. Although LUS has demonstrated high accuracy in diagnosing specific diseases [16], its effectiveness in pediatric ARF is currently undergoing clinical investigation [17,18]. A recent study indicated moderate agreement between CXR and LUS in diagnosing ARF in children when performed within six hours of each other, suggesting the need for further research to establish the definitive role of LUS in pediatric ARF cases [19].

Management of ARF often requires the application of a wide range of breathing support therapy, while simultaneously minimizing potential lung damage from therapeutic interventions [20]. Long-term outcomes can differ significantly; while some children fully recover without long-term consequences, others might suffer persisting respiratory issues, including chronic lung diseases, such as after PARDS.

This clinical review explores various aspects, from the pathophysiology underlying ARF in children to unique anatomical and physiological considerations and imaging techniques. Additionally, it sheds light on the emerging role of biomarkers and artificial intelligence. By exploring these topics, we aim to provide an updated overview of the clinical facets, with a particular emphasis on the diagnosis of pediatric ARF.

## 2. Materials and Methods

We searched for articles on PubMed and Google Scholar using the following search terms and logic for the introduction and general paragraphs on acute respiratory failure: “respiratory failure” OR “respiratory insufficient” OR “Acute Respiratory Distress Syndrome” AND “pediatric” OR “young” OR “infant” OR “children” OR “adolescents”. Additional search terms were adopted for each specific paragraph: for the pathophysiology and etiology “ventilation-perfusion mismatch” OR “surfactant dysfunction” OR “endothelial injury” OR “respiratory muscle fatigue” OR “alveolar damage”; for the diagnosis and monitoring paragraph “lung ultrasound” OR “chest x ray” OR “chest computed tomography” OR “chest magnetic resonance imaging” OR “radiographic assessment of lung edema score” OR “RALE” OR “end-tidal carbon dioxide monitoring” OR “arterial blood gases” OR “pulse oximetry”; for future perspectives paragraph “artificial intelligence” OR “machine learning” OR “predictive analytics” OR “personalized medicine” OR “biomarkers” OR “cytokine storm”. Additional studies were sourced from the references cited in select papers. Articles were selected according to their title and abstract, using the eligibility criteria. The inclusion criteria were: the English language; pediatric study population (age range 0–18 years); type of study: narrative and systematic reviews, longitudinal retrospective and prospective studies, and randomized control trials, including adult studies. Additionally, case reports, expert opinions, and manuscripts published in languages other than English were excluded. The final reference list was developed based on originality and relevance to the broader scope of this review.

## 3. Pathophysiology and Etiology

Pediatric ARF is a critical medical condition characterized by the sudden and severe inability of the respiratory system to maintain adequate oxygenation and carbon dioxide elimination.

Respiratory drive g is the result of a complex interaction between the lungs, the vascular and cardiac system, the brain, nerves, and muscles, which together cooperate to ensure adequate gas exchange [2].

Key factors that affect gas exchange include the following:

1. Adequate ventilation;
2. Gas concentration gradient between the alveoli and the bloodstream;
3. Respiratory membrane permeability;
4. Blood flow and transport of gases in blood.

**Figure 1:**
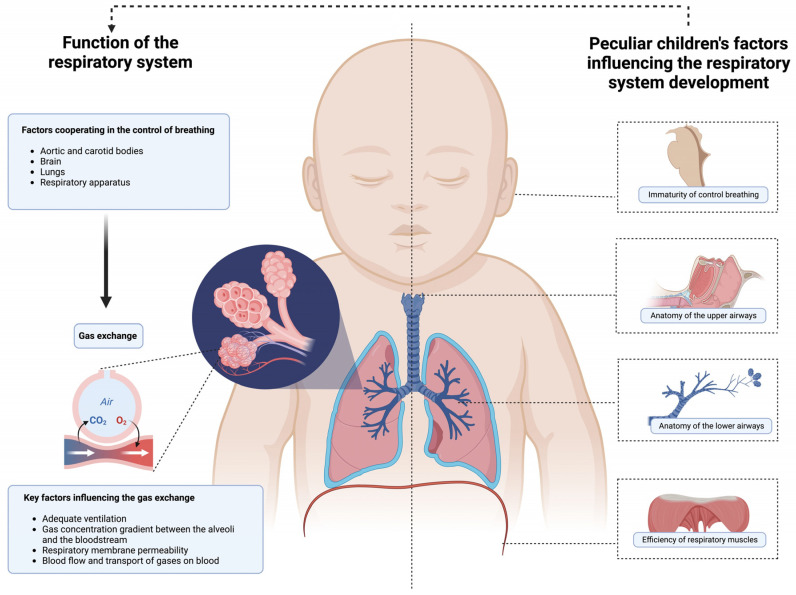
Pathophysiology of acute respiratory failure in infants and young children.

As illustrated in Figure 1, infants and young children can experience ARF more frequently than adults, due to a variety of unique factors including their developing respiratory system; the study of the anatomy and physiology of the respiratory system, in the pediatric field, is key to understanding the mechanisms underlying the pediatric increased susceptibility to ARF.

### 3.1. Respiratory Drive

Full development of respiratory control occurs during the final weeks of gestation and the first days after birth. Therefore, premature infants are more prone to respiratory distress from mild causes. Also, it is not uncommon to observe an irregular respiratory pattern during the initial days of life, due to a diminished response to hypoxia and hypercapnia [21].

### 3.2. Airways Anatomy

Nasal respiration performs fundamental functions of warming inspired air, filtering particulates and pathogens through mucosal and ciliary mechanisms, and providing an immunological barrier [22].

However, it is important to consider that the diameter of the nostrils and airways is generally reduced in children. Since airway resistance is inversely proportional to the airway radius, nasal obstruction and secretion management may be relevant factors in the development of acute respiratory insufficiency. Additionally, the musculature of the pharynx, larynx, and trachea exhibits greater collapsibility, making it more likely for airway obstruction to occur [23].

In the pediatric population, the number and volume of alveoli are lower than in adults; the connections between them permitting collateral ventilation, e.g., through Khon’s pores, are not fully developed in infants. Therefore, in cases of bronchial obstruction, atelectasis is more frequent, often resulting in ventilation/perfusion mismatch [24,25].

### 3.3. Respiratory Muscles

The horizontal orientation of the ribs and the diaphragm’s horizontal insertion make respiratory movements less effective in pediatric patients. Additionally, the reduced presence of fatigue-resistant type 1 muscle fibers results in greater susceptibility to fatigue during increased respiratory frequency, compared to adults, leading to a faster deterioration of breathing [22].

The control of breathing involves the synergy of the lungs, cardiovascular system, brain, nerves, and muscles to ensure optimal gas exchange. Gas exchange is influenced by several factors such as ventilation, gas concentration gradient, respiratory membrane permeability, and blood flow and transport. Infants and young children are more susceptible to ARF than adults due to a variety of peculiar aspects influencing the development of the respiratory system such as the immaturity of respiratory drive, airway anatomy, and respiratory muscle efficiency.

### 3.4. Etiology

**Figure 2:**
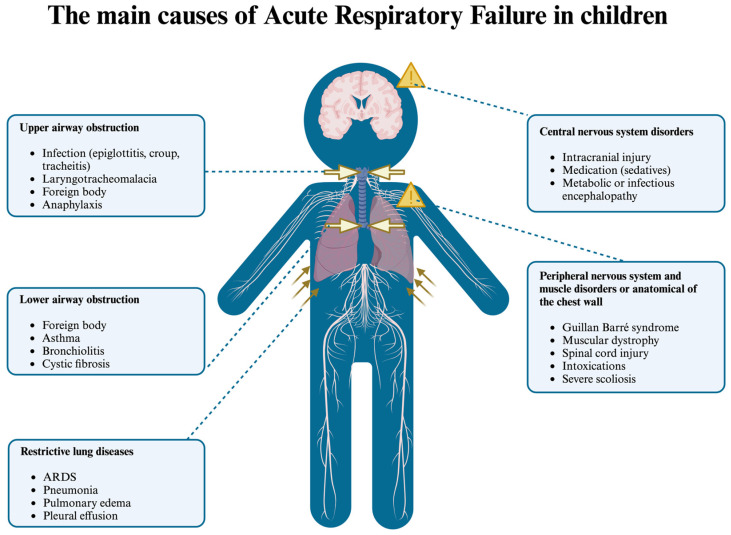
The main causes of ARF in children.

Common medical causes of ARF can be classified into the categories listed in Figure 2.

Cystic fibrosis, muscular dystrophy, and severe scoliosis may present as ARF but only in the frame of a concurrent viral or bacterial infection.

## 4. Diagnosis and Monitoring

The diagnosis of respiratory failure is primarily based on the patient’s clinical presentation and medical history. Clinical signs of common causes of ARF are summarized in Table 1.

Laboratory tests and instrumental examinations may support the assessment of the etiological cause underlying ARF and its severity. Two distinct clinical forms that may sometimes be confused are respiratory distress and ARF.

ARF is the pulmonary inability to ensure sufficient gas exchange to meet the body’s metabolic needs. Respiratory distress is a clinical definition and a compensatory condition in which the patient compensates for inadequate gas exchange by increasing respiratory rate and efforts, requiring prompt intervention [26]. PARDS is a peculiar form of ARF characterized by severe lung inflammation and injury, requiring specific diagnostic criteria. It is defined as affecting children without birth-related lung disease, with symptoms emerging within a week of a clinical event, characterized by lung edema not due to heart failure or fluid overload, chest imaging showing new lung opacities, and specific oxygenation levels for children on nasal respiratory support [27].

Thoracic inspection and auscultation are essential for clinical diagnosis. Notably, the sensitivity of lung auscultation is low compared with imaging tests [28].

### 4.1. Chest X-ray

Chest X-ray (CXR) is by far the most widely used imaging examination as a frontline tool in cases of ARF [16]. It is also the first radiological technique widely available at the bedside. The diffusion in hospitals of any level and its simplicity of execution are key elements, yet it requires a radiological service. CXR often provides critical information that aids in the rapid diagnosis of various conditions, although its use can sometimes be inappropriate [29]. In fact, despite guidelines advising against routine CXR for pediatric patients with mild and moderate asthma exacerbations and bronchiolitis, these radiographs are still frequently performed [30,31].

Pneumonia is a primary cause of ARF in the pediatric population. It is an infection of the lungs caused by bacteria, viruses, or fungi. Pneumonitis, on the other hand, is a more general term that refers to inflammation of lung tissue, which is not necessarily infectious. It is often caused by exposure to irritants such as chemicals, drugs, or radiation [32]. Although the diagnosis of pneumonia is clinical, based on the presence of persistent fever, increased respiratory rate, retractions indicating respiratory effort, and characteristic auscultatory sounds such as crackles, CXR is often requested as a confirmatory diagnostic test [33]. The Infectious Diseases Society of America and the Pediatric Infectious Diseases Society discourage the routine use of CXR in well-appearing children being evaluated for community-acquired pneumonia (CAP) in outpatient and emergency department (ED) settings [34].

In the current literature, few studies correlate the performance of CXR with the outcome of patients with pneumonia. Particularly, a retrospective cohort study involving 206,694 patients, demonstrated that the execution of a CXR reduces hospitalization by approximately 7 days for these patients [35]. On the other hand, a meta-analysis from 2018 highlighted that lung ultrasound has better sensitivity than CXR in diagnosing pneumonia in the pediatric population. Considering these results, improving ultrasound technique would bring significant advantages in diagnosing pneumonia, both in terms of diagnostic accuracy and in reducing radiation exposure to the patient [36].

In the case of ARF following an acute asthma attack, CXR is reserved for patients with severe manifestations and features suggesting the presence of complications (such as pneumothorax and pneumomediastinum), with the most common radiological abnormalities being: hyperinflation, segmental and subsegmental atelectasis, and pneumomediastinum (<1% of cases) [37]. Although complications associated with acute asthma attacks are not common, CXR is performed in 33–43% of patients, and the factors that most frequently drive the performance of this examination include the presence of fever, younger age, and an initial SpO_2_ < 91% [38].

CXR remains the diagnostic standard for two clinical conditions that often lead to ARF in the pediatric population: pneumothorax and foreign body inhalation.

Foreign body aspiration is a condition that pediatricians often encounter in the emergency department, and CXR represents the initial step in diagnosis. It helps recognize the position of the inhaled object and estimate its size, facilitating planning for subsequent invasive removal intervention through tracheobronchoscopy [39]. A challenge frequently faced is that these objects often appear radiolucent due to the material they are made of, necessitating the use of alternative investigative strategies [40].

Finally, in cases of suspected pneumothorax, CXR is the initial examination to request, allowing for visualization of small collections of air. The issue is that there is an absence of pediatric management guidelines, leading to controversial management strategies [41].

The primary challenge is assessing the risk of pneumothorax recurrence in the patient, which influences the decision regarding a more or less invasive approach. Many authors agree that pleural blebs are the main culprits of pneumothorax, and their presence is predictive of the recurrence of this clinical condition. Pleural blebs are better visualized through chest CT scans, so their current line of thought is consistent in recommending a chest CT scan for patients who experience multiple episodes of pneumothorax [42]. Conversely, other studies in the literature do not demonstrate a strict correlation between the presence of pleural blebs and the recurrence of the phenomenon, so they recommend performing a chest CT scan only in case of persistent air leaks in selected cases [43].

Guidelines on the approach and management of pneumothorax in pediatric patients could be helpful, for example, to standardize treatment and improve outcomes.

### 4.2. Lung Ultrasound

Lung ultrasound (LUS) is increasingly recognized as a valuable tool for assessing pediatric ARF. It offers numerous advantages, including portability, immediate results, and no radiation exposure, allowing for safe, bedside evaluations without the need to move critically ill children.

Firstly, it demonstrates promise for early risk stratification, with several studies exploring this application in bronchiolitis [44]. In that regard, the systematic literature review by Kogias et al. [44], highlighted the diagnostic and prognostic utility of LUS scores based on specific sonographic findings. The review analyzed 18 studies involving 1249 patients with bronchiolitis. Among them, four prospective studies successfully demonstrated the usefulness of LUS as a predictor of PICU admission [45,46,47,48]. Specifically, the LUSBRO score from the LUSBRO study, which assigned points from 0 to 3 to each of three pulmonary areas in both lungs based on LUS results for bronchiolitis, significantly outperformed scores based on clinical variables in predicting PICU admission. The criteria for PICU admission included the need for mechanical ventilation, non-invasive ventilation (NIV), a high-flow nasal cannula (HFNC) with a FiO_2_ over 60%, or apneas [45,49]. This study corroborated the results of Supino et al. [47] and Bueno-Campana et al. [50], who established a correlation between the LUS score and the need for HFNC or positive airway pressure support. These findings suggest that LUS, currently not included in the bronchiolitis guidelines, should be integrated into the initial diagnostic approach, thereby paving the way for future research.

Beyond bronchiolitis, LUS’s predictive role could be applied to other causes of pediatric ARF. A prospective study conducted by Giorno et al. [51] in the pediatric emergency department (ED), evaluated the potential of the LUS score as a prognostic tool for respiratory distress in children affected by various types of respiratory illnesses. They detected that an elevated LUS score upon admission was associated with a higher risk of requiring escalated care, such as HFNC, NIV, or mechanical ventilation, within 24 h.

Furthermore, LUS is a valuable prognostic and diagnostic tool in pediatric pulmonary conditions [52,53], including pneumonia. The detection of pneumonia through ultrasound has demonstrated high sensitivity and specificity in numerous studies [10]. Specifically, the randomized controlled trial by Jones et al. [43] involved 191 children suspected of having pneumonia in an ED. These children were divided into two groups: one underwent LUS with the option to follow up with CXR if needed, while the other received CXR followed by LUS. The results indicated a 38.8% reduction in CXR usage in the LUS-first group, with no missed cases of pneumonia in either group [54]. Moreover, LUS has proven effective in distinguishing between bacterial and viral pneumonia [55], in diagnosing pneumothoraxes in both neonates [56] and children [57], identifying spontaneous pneumomediastinum, and detecting pleural effusions [58].

Additionally, its effectiveness has been well-documented in acute neonatal pulmonary conditions, such as Respiratory Distress Syndrome (RDS) and Transient Tachypnea of the Newborn (TTN), which are the primary lung diseases encountered in neonatal ICUs [59]. In that respect, the study by Vergine et al. [60] aimed to assess the diagnostic accuracy of LUS by comparing its outcomes with clinical diagnoses, which served as the benchmark. The clinical diagnoses were determined by a single neonatologist who was not involved in the LUS procedures and did not use ultrasound data. CXR was included in the workup when deemed necessary by the attending physician. The findings showed that LUS correctly identified TTN in 28 out of 30 cases, and RDS in 22 out of 23 cases. In neonatal ICUs, LUS application is more advanced and widespread compared to PICUs, although its adoption still varies widely and lacks an international guideline [61].

The main ultrasound findings in pediatric ARF are presented in Table 2.

While the application of LUS in discerning undifferentiated respiratory distress etiologies in children has been explored in various ED settings, research on its efficacy in the PICU remains limited. DeSanti et al. [62] conducted the first blinded Point-of-Care Ultrasound (POCUS) examination in children admitted to the PICU with pneumonia, bronchiolitis, and status asthmaticus. The study revealed moderate sensitivity and specificity, in contrast to findings in adults, where POCUS proves highly effective in discerning causes of ARF [13]. The same group also studied LUS artifacts in 87 children with ARF and discovered significant heterogeneity and overlap in POCUS artifacts [63]. Another study was conducted to evaluate the diagnostic interpretation agreement of POCUS between pediatric intensivists and an expert intensivist in children with ARF in the PICU, finding moderate agreement [64]. Overall, these studies emphasize the challenges of standardizing image interpretation and underscore the necessity for comprehensive training and clear guidelines to enhance the reliability and effectiveness of LUS in pediatric critical care. This approach could be exceedingly beneficial in PICUs, where LUS is notably advantageous for providing rapid, non-invasive assessments directly at the patient’s bedside. This capability is essential for repeated evaluations, particularly for patients in unstable conditions who require prompt, evidence-based decisions.

In summary, there is a relevant need for ongoing research and the integration of guidelines to unveil the potential of LUS in pediatric acute care settings.

### 4.3. Chest Computed Tomography

Computed tomography (CT) is the gold standard in thoracic imaging in adults. In children, CT, despite it is high accuracy, is a second-line diagnostic tool, when initial clinical assessments and conventional radiography and/or LUS do not provide definitive results. With high spatial resolution, CT provides an unparalleled view of pulmonary structures, excelling in detecting and characterizing critical lung abnormalities underlying pediatric respiratory emergencies [65]. Notably, recent advancements in CT technology, such as helical and multi-detector CT (MDCT), have enhanced image quality and reduced scanning times, which are crucial in acute settings [66].

Despite its comprehensive diagnostic capabilities, CT is generally considered a secondary option due to concerns about radiation exposure in children [67]. The use of CT is guided by strict clinical protocols that emphasize the ‘as low as reasonably achievable’ (ALARA) principle, aiming to minimize scan frequency and reduce radiation doses to the lowest possible levels [68]. Guidelines typically recommend CT scans in acute pediatric respiratory distress when other imaging modalities are inconclusive but the clinical suspicion of a severe underlying condition remains high, there is a suspicion of anatomic abnormalities, and when there are specific indications like severe trauma or suspected foreign body aspiration [69].

CT in pediatric ARF is useful for identifying serious pneumonia complications not typically visible on standard CXR or LUS, such as abscesses, bronchopleural fistulas, and cavitary necrosis [70]. Moreover, in immunocompromised children suspected of having pneumonia, chest CT is crucial for rapidly diagnosing infections and differentiating them from other lung conditions, such as bronchiolitis obliterans and alveolar hemorrhage [71].

Furthermore, CT is beneficial in acute presentations of congenital lung abnormalities, such as congenital lobar emphysema, congenital pulmonary airway malformation, bronchopulmonary foregut malformation, lung agenesis and aplasia, congenital pulmonary venolobar syndrome (also known as Scimitar syndrome), and horseshoe lung [72].

If pulmonary embolism or infarction is suspected, CT, and especially CT angiography, is indispensable for visualizing the pulmonary vasculature in detail [73].

For trauma, CT is invaluable for a comprehensive evaluation of injuries that may manifest as respiratory failure, including hemothoraces, lung contusions, and airway injuries, which are vital for planning immediate and effective interventions [74].

Although rare, CT may be used to investigate suspected foreign body aspiration in cases where bronchoscopy poses high risks or has yielded inconclusive and non-diagnostic results [75].

From a clinical point of view, if a patient’s condition does not improve or worsens, CT can reveal underlying conditions or complications that were not initially apparent, guiding more targeted treatment strategies. Therefore, ensuring the judicious use of CT in these scenarios is crucial, balancing the urgent need for detailed pulmonary imaging against the necessity to protect pediatric patients from unnecessary radiation exposure.

### 4.4. Lung Magnetic Resonance

Nuclear magnetic resonance (MRI) for the diagnosis of respiratory pathologies in pediatric settings is intriguing yet mainly used for research purposes. A strength of MRI is to avoid the potential radiation damage caused by CXR and chest CT scans, which are of great concern in the pediatric population due to the increased radiosensitivity of tissues and the longer time horizon to manifest consequence [76].

However, there are limitations to consider in the use of pulmonary MRI [77] including the following:

- Thoracic organs are moving structures during breathing with a reduced signal-to-noise ratio;
- Pulmonary parenchyma has a low density of protons;
- The presence of numerous air–tissue interfaces leads to the formation of many magnetic field gradients, causing significant signal phase shifts and geometric distortions;
- The longer image acquisition times and the need for breathing instructions, may require the use of sedation in the pediatric population.

New techniques and protocols in MRI allow us to overcome these limitations. Among them, there is the ultra-short echo sequences MRI (UTE-MRI), which minimizes artifacts and enables more accurate visualization and distinction of both hyperintense and hypointense structures [78].

Currently, MRI is utilized in some centers in cases of ARF associated with complicated clinical presentations [79].

MRI exhibits high specificity in detecting the presence of lung abscesses or lesions in the central parenchyma. The sensitivity of MRI is reported to be 90% in recognizing lung consolidations [80]. Additionally, its sensitivity reaches 75% in discriminating bronchiectasis, and its specificity is suggested to be high, approaching the high specificity seen with CT scans [81,82].

Additionally, another category in which MRI finds application is the study of complications associated with cystic fibrosis, providing crucial information about the presence of mucus, the thickness of bronchial walls, areas of consolidation, and the presence of segmental or lobar destruction [83].

Lastly, MRI is of potential clinical relevance in selected cases of ARF in the neonatal population, as it can identify congenital malformations underlying the onset of the acute condition, such as pulmonary and upper airway malformations, segmental bronchial atresia, bronchopulmonary sequestration, congenital lobar overinflation, and the presence of bronchogenic cysts [80].

Among the neonatal population, patients with bronchopulmonary dysplasia warrant special consideration, as they often experience ARF. This occurs both due to the reduced alveolar–capillary exchange surface area and the increased collapsibility of the airways [84].

Recent studies have shown that MRI can assess the degree of collapsibility of the airways, and consequently, it can provide insights into estimating the risk of developing ARF. Bates et al. [85], through studying the changes in tracheal diameter during inspiration and expiration, have demonstrated that the collapsibility of this structure is significantly greater in patients with moderate to severe bronchopulmonary dysplasia.

In summary, MRI is not currently used as a frontline diagnostic tool for acute respiratory failure. However, it plays an increasingly significant role in diagnosing specific conditions that often underlie this pathology, and it may have a more important role in the future.

### 4.5. Monitoring

Monitoring is essential in ARF to comprehend the degree of the response to the therapy administered, to detect complications, and to predict outcomes.

Therefore, it is necessary to continually assess the clinical course through physical examination, continuously monitor vital parameters using a pulse oximeter, non-invasively assess blood CO_2_ levels through capnography, and precisely monitor gas exchanges invasively through arterial blood gas analysis [86].

Arterial blood gas analysis (ABG) is pivotal in ARF as it allows for the identification of PaO_2_ and PaCO_2_ values. This enables the differentiation between hypoxic ARF (PaO_2_ < 60 mmHg), hypercapnic ARF (PaCO_2_ > 45 mmHg), or mixed respiratory failure. However, there are some limitations, particularly in children [87]. ABG is an invasive procedure and obtaining an arterial blood sample in pediatric patients can be challenging. It is essential to note that in cases of ARF, ABG must be performed repeatedly to monitor the patient’s clinical progression [88].

Venous sampling, which is easier, is not as reliable as arterial sampling [86].

A pulse oximeter is consistently utilized to evaluate respiratory status by providing real-time, non-invasive monitoring of a patient’s oxygen saturation levels. Yet in cases of peripheral vasoconstriction, peripheral edema, sweating, and significant limb movements, the pulse oximeter may provide inaccurate readings due to impaired signal acquisition [89]. For clinical purposes, oxygen saturation (SpO_2_) values < 94% may indicate hypoxia [90,91].

Furthermore, capnographs, employing infrared CO_2_ absorption capabilities, are primarily employed in intubated patients to assess potential increases in dead space or identify obstructions of the endotracheal tube.

The initial step is to assess the cardiorespiratory stability of the patient using the conventional “ABCD” flowchart.

Subsequently, it is imperative to evaluate the severity and type of ARF by performing an ABG. However, given the time-sensitive nature of these situations, assessing the degree of hypoxia through SpO_2_ measurement often becomes a more practical approach.

## 5. Future Perspectives

### 5.1. The Role of Biomarkers

Biomarkers play a crucial role in modern medicine, offering measurable indicators of pathologies that can significantly enhance diagnostic accuracy, guide treatment decisions, and predict clinical outcomes. Acute lung disease and mechanical ventilation (MV) damage the alveolar–capillary barrier, increasing its permeability and resulting in protein leakage and the spillover of inflammatory mediators into circulation [92]. Some studies in pediatrics have investigated the use of biomarkers to assess the risk of progression and the severity of ARF allowing for timely and targeted interventions [93,94]. However, pediatric research in this field is expanding upon initial studies conducted in adults [95,96], although it remains limited [97].

Respiratory Syncytial Virus (RSV) is a common cause of ARF in neonates and infants. Research has explored how certain biomarkers are linked to the severity of RSV-related bronchiolitis and bronchopneumonia. Specifically, higher blood levels of surfactant protein D (SP-D) and Krebs von den Lungen-6 (KL-6) have been associated with more severe lung damage [98,99]. Additionally, specific cytokines like IL-8, IL-6, and IFN-γ [100,101,102], as well as the nasopharyngeal microbiome composition, including the presence of bacteria such as Moraxella and Haemophilus [103], have also been linked to the severity of inflammation. However, as highlighted by a systematic literature review by Öner et al. [104], there are significant gaps in finding reliable biomarkers for clinical purposes.

As far as pediatric ARF is concerned, a marker that currently appears to provide a negative prognostic role is serum thrombomodulin (sTM), an anti-thrombotic agent located on the surface of endothelial cells, that is converted into its soluble form when local endothelial damage occurs [105].

In an ancillary study of the multicenter clinical trial RESTORE [106], plasma thrombomodulin values were evaluated in 432 patients within 5 days after intubation [107]. Monteiro et al. demonstrated that high initial levels of sTM, particularly on the first day, are associated with multiorgan failure and increased risk of 90-day mortality. Furthermore, elevated sTM levels were significantly correlated with worsening oxygenation index in the first 5 days after intubation, suggesting that pulmonary vascular damage is a principal contributor to serum sTM. Therefore, these results underscore the potential role of vascular injury in the pathogenesis of ARF and the prognostic value of sTM as a biomarker for targeted therapies. These findings are consistent with those of the previous study by Orwoll et al. in the field of PARDS, which found that elevated sTM levels were associated with increased organ dysfunction and mortality in children with ARDS [108]. Additionally, plasminogen activator inhibitor-1 (PAI-1) is a biomarker of antifibrinolysis and vascular endothelial dysfunction, which is associated with higher mortality in pediatric ARF [93]. Most recently, the Radiographic Assessment of Lung Edema (RALE) score, which summarizes the extent and density of chest X-ray infiltrates, has shown associations with plasma biomarkers and clinical outcomes in children with ARF [94].

In ARDS, circulating biomarkers have been extensively studied in adult patients [96], yet there are fewer data on children. A prospective observational cohort study aimed to identify a panel of biomarkers, sampled in BAL, capable of evaluating the severity and risk of evolution of ARDS [109].

This is a path widely followed in the adult population, however, it has not been taken into consideration due to the extreme heterogeneity of the pathologies that may underlie ARF.

On the contrary, in the pediatric population, the causes of ARF are mainly infectious, thus it would be much easier to find specific markers in the BAL to evaluate the risk of worsening. However, very few studies have been conducted in this area.

From one of these studies, an increase in CCL7 (C-C motif chemokine ligand 7) emerged as the only indicator of increased severity of pediatric ARDS. Due to the small size of the sample analyzed, this result was compared with that obtained from mice, in which an increased level of CCL7 was found in mice affected by lethal influenza type A. Furthermore, the lack of CCL7 in response to LPS administration suggests its role as a marker of viral infection [110].

Another study by Zinter et al. [111] identified two distinct matrix metalloproteinase (MMP) profiles in 235 children with PARDS. Profile 1, which had elevated levels of several MMPs including MMP-1, -2, -3, -7, -8, TIMP-1, and TIMP-2, and lower levels of MMP-9, was linked to higher inflammation, endothelial injury markers, and worse clinical outcomes. Profile 2 had lower levels of these MMPs. These findings suggest that MMP profiles could be used to predict disease severity and guide targeted therapies in pediatric ARDS.

A more recent study investigating the use of plasma biomarkers to identify subphenotypes in PARDS [112] analyzed 19 biomarkers in 279 children over seven days. Researchers identified distinct biomarker patterns between survivors and non-survivors and found that elevated levels of tissue injury markers and inflammatory cytokines were more strongly associated with multiple organ dysfunction syndrome (MODS) rather than persistent ARDS, underscoring the potential of using biomarker profiles to tailor therapeutic approaches. These findings align with the previous study by Dahmer et al. [113], which identified two distinct phenotypes in 304 children with PARDS: one hypoinflammatory (phenotype 1) and one hyperinflammatory (phenotype 2). The hyperinflammatory phenotype was associated with higher concentrations of inflammatory biomarkers, increased use of vasopressors, and a greater incidence of sepsis. Additionally, phenotype 2 demonstrated worse clinical outcomes, including prolonged mechanical ventilation and increased mortality.

The identification of valid and easily measurable biomarkers could be a turning point in clinical management. Biomarkers could be particularly helpful in the early stratification of patients based on the risk of progression, allowing for timely and appropriate intervention tailored to the patient’s initial condition.

However, it seems clear that we are still far from outlining a panel of biomarkers to evaluate and stratify the severity of patients with ARF. The studies conducted so far, however, seem to argue in favor of a future in which ARF could be treated in the most personalized way possible, thanks to the help of specific biomarkers.

### 5.2. Artificial Intelligence

Artificial intelligence (AI) stands as a swiftly evolving technological frontier, offering both promises and challenges in the field of medical diagnostics [114]. The potential uses of AI in diagnosing ARF are extensive, as emphasized by Zhang and Wittenstein’s invitation to research AI’s role in revolutionizing ARF management through improved diagnostics and personalized treatments [115].

In imaging analysis, although many AI tools are initially designed for adults [116], their adaptation to pediatric care is underway [117], providing heterogeneous results. Some studies indicate that AI can achieve radiologist-level accuracy in detecting pneumonia from CXRs [118,119].

However, it is important to note that AI systems trained on adult data may not perform as effectively when applied to children. For instance, a study by Shin et al. [120], which analyzed 2273 pediatric chest X-rays (CXR), found that the accuracy of AI-based software originally developed for adults varied by age. Specifically, younger children, particularly those under two years of age, were more likely to receive incorrect diagnoses. This underscores the need for further validation and adaptation of AI for younger pediatric populations.

Another application of AI in the early detection and diagnosis of respiratory conditions contributing to ARF involves the recognition of pathological pediatric breath sounds. In this respect, the study by Kevat et al. [121] evaluated an AI algorithm’s ability to generalize detection using recordings from digital stethoscopes. After analyzing 192 children’s recordings, AI demonstrated high accuracy in identifying crackles and wheezes. Each recording was validated by human experts before AI analysis to ensure accuracy, confirming that AI could adapt its detection capabilities across different devices and demonstrating significant potential for broader clinical use.

The application of AI in NICUs and PICUs, as discussed in the systematic review by Adegboro et al. [122], remains primarily a research domain. This review highlights AI’s role in enhancing diagnostic accuracy by interpreting complex medical data, aiding in the early diagnosis of conditions such as sepsis and respiratory disorders. This capability may improve the speed of diagnosis and enhance precision, potentially leading to better-tailored and timely interventions that can significantly affect patient outcomes. A recent study in the PICU setting employed machine learning models to assess the predictive power of various biomarkers for mortality in patients with respiratory diseases [123]. By analyzing a large dataset of 13,944 ICU admissions, the researchers identified crucial biomarkers, such as lactate, pCO_2_, LDH, and creatinine. The study highlighted the effectiveness of advanced machine learning techniques, achieving an area under the curve (AUC) score of 85.2% and an accuracy of 89.32% in predicting mortality. However, this area of AI application is still evolving, with ongoing research required to address challenges in data diversity, potential biases, and clinical workflow integration.

Beyond diagnostics, recent advancements further illustrate the potential of AI in managing ARF. AI-driven systems can analyze continuous data from mechanical ventilation parameters to provide insights difficult to achieve through traditional methods [124]. This continuous monitoring could facilitate the early detection of complications and optimize ventilation strategies. Studies on adult populations have shown that AI can be valuable in predicting weaning outcomes [125,126], but similar validations in pediatric cohorts are needed to confirm these benefits for younger patients. While AI shows promise in predicting outcomes and aiding in ARF management, significant limitations exist [127]. For instance, AI systems require large, high-quality datasets to function accurately, and there is a risk of bias if these datasets are not representative of diverse patient populations [128]. Without addressing the ongoing concerns about AI [129], our view is that continuous high-quality research is essential to ensure the effectiveness, safety, and reliability of AI systems, aiming to improve diagnostics and patient outcomes.

## 6. Limitations of the Study

Some methodological concerns need to be addressed. This is a narrative review and as such, it has its limitations. Firstly, a narrative review is often more subjective, relying heavily on the authors’ expertise and perspective, which can introduce bias in the selection and interpretation of studies. Secondly, the methodology is generally less rigorous, lacking a standardized protocol for searching, selecting, and appraising the literature. Lastly, due to the lack of a systematic approach, it is often less reproducible, with different reviewers potentially arriving at different conclusions based on the same body of literature [130]. However, the present work provides a broad and more flexible overview of this topic, identifying research gaps, and informing future studies.

## 7. Conclusions

Various clinical conditions may provoke ARF in children. Its assessment is challenging due to children’s compliance and diagnostic difficulties in confirming and estimating the severity of the ARF and defining the diagnosis. Precise visualization with lung imaging is of utmost importance, and prompt support, such as with using mechanical ventilation in severe cases, is essential to support the treatment of the underlying disease. Here, we also reported current research on interesting biomarkers and insights in AI. In both these frontier fields, high-quality research in children is essential to ensure significant improvements in diagnostics and patient outcomes.

## Figures and Tables

**Table 1:** Clinical presentation of major causes of ARF.

Asthma exacerbation	Wheezing, difficulty breathing, chest tightness, cough, and use of accessory muscles
Bronchiolitis	Rapid breathing, wheezing and crepitations, cough, nasal flaring, retractions, and use of accessory muscles
Pneumonia	Rapid breathing, fine crepitations, fever, increased respiratory rate, difficulty breathing, use of accessory muscles, and retractions
Foreign Body Aspiration	Sudden onset of coughing, choking, wheezing, unilateral breath sounds reduction or wheezing, and possibly cyanosis
Epiglottitis	Severe sore throat, fever, drooling, difficulty swallowing, muffled voice, and stridor
Pulmonary Edema	Tachypnea, respiratory distress, cyanosis, and rales or crackles on auscultation
Septic Shock	Rapid breathing, altered mental status, fever or hypothermia, signs of poor perfusion, warm extremities
Anaphylaxis	Sudden respiratory distress, wheezing, possible stridor, skin rash, facial swelling, and shock

**Table 2:** The main lung ultrasound findings in pediatric ARF.

LUS Finding	Description	Bronchiolitis	Pneumonia	PTX	PARDS
Air Bronchograms	Branching structures within a consolidation	Frequently observed in Consolidation	Classic finding in bacterial pneumonia	Absent	Seen in dense consolidation
A-lines	Horizontal lines parallel to the pleural line	Present if areas are not severely affected	Reduced or absent in areas of consolidation	Normal pattern away from PTX area	Reduced or absent in areas of consolidation
B-lines (Comet Tails)	Hyperechoic vertical lines from the pleural line	Numerous and Widespread	Variable, depending on severity and complications	Absent over the area of PTX	Numerous, interstitial edema Diffuse or patchy distribution
Consolidation	Hypoechoic areas within the lung	Common in severe cases	Typical finding Lung hepatization, may show dynamic air bronchogram	Absent	Patchy or diffuse, common
Lung Sliding	To-and-from movement of the pleural line	Usually present unless complicated by PTX	Usually present unless complicated by PTX	Absent over the PTX	Usually present unless complicated by PTX
Pleural Line Abnormalities	Irregular, thickened, or fragmented pleural line	Possible irregularities	Common, indicating pleural involvement	Sudden absence of pleural line at PTX site	Often abnormal due to inflammation
Pleural Effusion	Anechoic space between pleural layers	Uncommon in Bronchiolitis	Common in complicated cases	May occur secondary to trauma or infection	Can occur in severe cases
